# The impact of reimbursed Primary Psychological Care in Belgium.

**DOI:** 10.1192/j.eurpsy.2026.10773

**Published:** 2026-07-31

**Authors:** C. Kubicek, R. Bruffaerts, L. Jansen, F. Glowacz

**Affiliations:** 1 Center for Public Health Psychiatry, KU Leuven, Leuven; 2 Université de Liège, Liège, Belgium

## Abstract

**Introduction:**

Since the beginning of this century, Belgium has been going through important shifts and reforms regarding the organization of mental healthcare. One of the most important shifts lays in the de-institutionalization of care to a more community-based mental healthcare. These reforms are embedded in a public health approach. Building on these changes, Belgium established in 2019 a system of reimbursed primary psychological care (PCP), providing low-intensity and low-threshold interventions, both individually or in groups.

**Objectives:**

This study aims at identifying which patients make use of PCP (1) and the effect of the intervention on clinical outcomes on the patient-level (2).

**Methods:**

A longitudinal study was performed. N=2,745 patients treated with PCP were included at baseline and followed-up at 3, 6 and 12 months. The instrument includes questions on socio-demographics, mental disorders and related problems, quality of life, previous treatment use and treatment barriers.

**Results:**

Over 70% of the patients met criteria for a DSM-5 mental disorder, both for lifetime and 12 months prevalence, with anxiety and depression as the most frequently identified disorders. Suicidal ideation and behaviors within the last 12 months were reported by more than one-third of patients. 43.9% had never engaged with mental health services prior to PCP treatment. The median duration between symptom onset and initial treatment contact was 4 years. Patients improved positively on symptomatology, quality of life and resilience. A commonly cited barrier to seeking professional help was the belief that the issue could be managed independently.

**Conclusions:**

Up to 2025, more than 500 000 patients made use of PCP. The intervention covered a large number of patients with varying pathologies and severity. Most importantly this intervention was able to shorten the delay of seeking treatment to 4 years compared to 10 years in the Belgian general population. Furthermore, patients treated with PCP showed improvement on several clinical outcomes, further underpinning it’s positive societal and economic impact. Next to a continuous longitudinal clinical and epidemiological study, future research will focus on the nature of the interventions proposed in PCP and a thorough socio-economic analysis.

**Disclosure of Interest:**

None Declared

